# Patterns of Chinese medicine use in prescriptions for treating Alzheimer’s disease in Taiwan

**DOI:** 10.1186/s13020-016-0086-9

**Published:** 2016-03-28

**Authors:** Shun-Ku Lin, Sui-Hing Yan, Jung-Nien Lai, Tung-Hu Tsai

**Affiliations:** Department of Chinese Medicine, Taipei City Hospital, Ren-Ai Branch, Taipei, Taiwan; Department of Neurology, Taipei City Hospital, Ren-Ai Branch, Taipei, Taiwan; The Graduate Institute of Integrated Medicine, China Medical University, Taichung, Taiwan; School of Chinese Medicine, China Medical University, Taichung, Taiwan; Institute of Traditional Medicine, School of Medicine, National Yang-Ming University, Taipei, Taiwan

## Abstract

**Background:**

Certain Chinese medicine (CM) herbs and acupuncture may protect against Alzheimer’s disease (AD). However, there is a lack of research regarding the use of CM in patients with AD. The aim of this study was to investigate CM usage patterns in patients with AD, and identify the Chinese herbal formulae most commonly used for AD.

**Methods:**

This retrospective, nationwide, population-based cohort study was conducted using a randomly sampled cohort of one million patients, selected from the National Health Insurance Research Database between 1997 and 2008 in Taiwan. CM use and the top ten most frequently prescribed formulae for treating AD were assessed, including average formulae dose and frequency of prescriptions. Demographic characteristics, including sex, age and insurance level were examined, together with geographic location. Existing medical conditions with the behavioral and psychological symptoms of dementia, and medications associated with CM were also examined. Factors associated with CM use were analyzed by multiple logistic regressions.

**Results:**

The cohort included 1137 newly diagnosed AD patients, who were given conventional treatment for AD between 1997 and 2008. Among them, 78.2 % also used CM treatments, including Chinese herbal remedies, acupuncture and massage manipulation. Female patients (aOR 1.57 with 95 % CI 1.16–2.13) and those living in urban areas (aOR 3.00 with 95 % CI 1.83–4.90 in the middle of Taiwan) were more likely to use CM. After adjusting for demographic factors, AD patients suffering from the behavioral and psychological symptoms of dementia were more likely to seek CM treatment than those with no symptoms (aOR 2.26 with 95 % CI 1.48–3.43 in patients suffering more than three symptoms). *Bu*-*Zhong*-*Yi*-*Qi*-*Tang* and *Ji*-*Sheng*-*Shen*-*Qi*-*Wan* were the two formulae most frequently prescribed by CM practitioners for treating AD.

**Conclusion:**

Most people with AD who consumed herbal products used supplement *qi*, nourish the *blood*, and quiet the heart spirit therapy as complementary medicines to relieve AD-related symptoms, in addition to using standard anti-AD treatments.

**Electronic supplementary material:**

The online version of this article (doi:10.1186/s13020-016-0086-9) contains supplementary material, which is available to authorized users.

## Background

Alzheimer’s disease (AD) is a neurodegenerative disease characterized by substantial memory impairment, aphasia, apraxia, agnosia and function disturbance [1]. AD is the most common cause of dementia in older people and accounts for 60–80 % of reported dementia cases in the World [2]. Although psychosocial and pharmacologic interventions can be effective for alleviating AD-related symptoms [3], there is currently no curative treatment for AD.

It has been reported that some Chinese herbs may protect brain cells against AD [4, 5]. However, clinical evidence regarding the efficacy of Chinese medicine (CM) for treating AD is limited. This study therefore aims to investigate the extent to which CM is used by patients suffering from AD, and to identify the most common Chinese herbal formulae used by AD patients to treat their symptoms.

## Methods

This study was approved by the Taipei City Hospital Institutional Review Board under study number TCHIRB-1020816-E (Additional file 1) and National Health Research Institutes under study number NHIRD-103-091 (Additional file 2). The research and associated reporting have been conducted in accordance with conventional ethical principles to protect the identities of the patients involved.

### Data resources

This retrospective, nationwide, population-based cohort study was conducted using a randomly sampled cohort of one million patients, selected from the National Health Insurance Research Database (NHIRD) between 1997 and 2008 in Taiwan. The National Health Insurance (NHI) system covers over 97 % of the population of Taiwan and provides full reimbursement to its members for treatments based on CM, including Chinese herbal products, acupuncture, moxibustion and traumatology manipulative therapies. The NHI also holds all of the medical records for CM treatments prescribed by licensed CM practitioners [6].

One million patient records were randomly selected from those of the 23 million Taiwanese people currently insured under the NHI system. No significant differences were observed in the demographic distribution between the random sample and the original NHIRD. The medical records used in this study were collected for the dates between the 1st of January, 1996 and the 31st of December, 2008. The NHIRD database contained complete data pertaining to clinical visits, and hospitalization records, including visit date, hospital, physician specialist, major diagnoses and the dosage and frequency of any medicines prescribed. The Bureau of National Health Insurance requires physicians to record major diagnoses according to the format described in the International Classification of Diseases, Ninth Revision, Clinical Modification (ICD-9-CM).

### Study sample

**Fig. 1:**
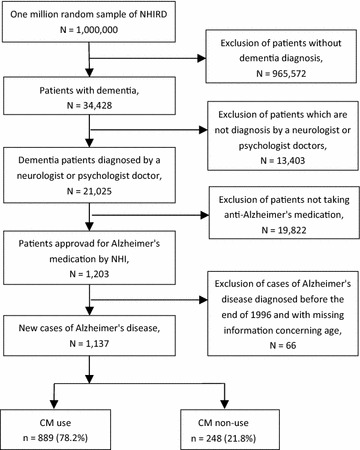
Flow chart detailing the recruitment of subjects from random sample of one million patients from the NHIRD between 1997 and 2008

Figure 1 shows a flowchart describing of the sample selection process. First, all patients without dementia (ICD-9 codes: 290.0, 290.1, 290.2, 290.3, 290.4, 294.1, 294.2 and 331) were excluded (n = 965,572). Second, we excluded patients with a dementia diagnosis not made by a qualified neurologist or psychologist (n = 13,403), along with patients that did not receive anti-AD treatment (n = 19,822). Doctors in Taiwan are requested by the Bureau of National Health Insurance to only prescribe anti-AD drugs to patients that fulfill the criteria outlined by the National Institute of Neurological and Communicative Disorders and Stroke (NINCDS), the Alzheimer’s Disease and Related Disorders Association (ADRDA) or the Diagnostic and Statistical Manual of Mental Disorders (DSM IV-TR). Finally, cases of AD diagnosed before the end of 1996 were excluded from the current study, as well as those without complete NHI reimbursement data (n = 66) to ensure that all of the patients represented newly diagnosed cases of AD. Upon completion of this exclusion process, we had a study cohort consisting of 1137 patients.

### Study variables

Several demographic factors were selected from previous studies [7]. The patients were categorized into six different age groups: <45, 45–54, 55–64, 65–74, 75–84 and ≥85 years of age. The geographic locations of the patients were sorted into seven different regions with similar regional environments: Taipei City and Kaohsiung City, the Northern, Central, Eastern and Southern regions of Taiwan, and the outlying islands.

The amount of money to which each patient was insured was divided into four levels: 0, 1–19,999, 20,000–39,999 and >40,000 new Taiwanese dollars. The “0” insured level included unemployed and retired patients, for whom the insurance costs were covered by the government or other family members.

The NHIRD database was searched for diagnosis and treatment records relating to the behavioral and psychological symptoms of dementia (BPSD), including delirium (ICD9 codes: 290.11, 290.3 and 290.41), delusions (ICD9 codes: 290.20, 290.12 and 290.42), depression (ICD9 codes: 290.13, 290.21 and 290.43), sleep disturbances (ICD9 codes: 780.5 and 307.4), hallucination (ICD9 codes: 7801, 2913 and 2928) and behavioral disturbances (ICD9 codes: 294.11 and 294.21). The reimbursement database contains all of the important details relating to the prescription of conventional medicines for treating AD. The types of prescription drugs used to treat AD were categorized as follows: donepezil, rivastigmine, galantamine, memantine, deprenyl, antipsychotic, antimanic, anxiolytic and antidepressant.

The comorbidities of the patients were categorized using the icd-9-cm codes. Notably, the results of previous studies have shown that some comorbidities, including diabetes mellitus (icd9 codes: 249 and 250), hypertension (icd9 codes: 401–405), hyperlipidemia (icd9 codes: 272), chronic kidney disease (icd9 codes: 585, 586 and 588), cerebral vascular accident (icd9 codes: 430–438) and atherosclerosis (icd9 codes: 440), can affect the prognosis and treatment of patients diagnosed with AD.

### Chinese medicine treatment

Chinese herbal products (CHPs) composed of one or more herbs (i.e., herbal formulae), which are covered by the NHI system, are more widely adopted by Taiwanese patients than any other form of CM. [8] Information pertaining to CHPs was obtained from the Department of Chinese Medicine and Pharmacy (DCMP), Ministry of Health and Welfare, Taiwan, including the name and ingredients of their herbal formula, as well as their pharmaceutical effects, DCMP manufacturing code and manufacturer. CHPs with the same good manufacturing practices and DCMP standards were sorted into the same group [9]. The acupuncture therapy including acupoint and frequency and massage manipulation and treatment position were also record by the NHI system.

### Statistical analysis

The factors associated with the CM treatments used in the current study were analyzed using multiple logistic regression with a significance level of α = 0.05. An odds ratio was used to quantify the relationships between the different conditions and CM use. All of the odds ratios were adjusted based on the demographic factors described above. Version 9.3 of the SAS statistical analysis software (version 9.4; SAS Institute Inc., Cary, NC, USA) was used for data management and analysis.

## Results

The NHIRD of outpatient claims contained information pertaining to the claims of 1137 patients with AD from 1997 to 2008. Among them, 889 (78.2 %) AD patients used CM outpatient services. The mean age of non-CM users was slightly higher than that of CM users.

Table 1 shows details concerning the demographic and medication distributions of the CM and non-CM users. After adjusting for demographic factors, the results clearly showed that AD patients suffering from one or more BPSD (one symptom: OR = 0.90, 95 % CI 0.57–1.44; two symptoms: OR = 1.94, 95 % CI 1.24–3.05; three or more symptoms: OR = 2.26, 95 % CI 1.48–3.43) were more likely to seek CM treatment than those with no BPSD. Female patients and those living in urban areas were more likely to seek CM treatment than male patients and those living in rural areas. No discernible difference was observed in CM use between AD patients suffering from one or more comorbidities (one: OR = 1.1, 95 % CI 0.47–2.58; two: OR = 0.91, 95 % CI 0.41–2.01; and three or more types: OR = 1.76, 95 % CI 0.83–3.74) and those with no comorbidity.

**Table 1:** Demographic and medication characteristics and results of multiple logistic regression for CM use among patients with Alzheimer’s disease

Characteristics	CM^a^ users	CM^a^ non-users	aOR^b^ (95 % CI^c^)
No. of cases	889 (78.2 %)	248 (21.8 %)	
Age at diagnosis (incidence rate)			
<45 (0.001 %)	7 (0.8 %)	2 (0.8 %)	0.78 (0.15–4.05)
45–54 (0.036 %)	27 (3 %)	2 (0.8 %)	3.64 (0.80–16.53)
55–64 (0.246 %)	115 (12.9 %)	27 (10.9 %)	1
65–74 (1.185 %)	309 (34.8 %)	92 (37.1 %)	0.74 (0.45–1.22)
75–84 (7.105 %)	381 (42.9 %)	107 (43.1 %)	0.83 (0.51–1.36)
≥85 (17.942 %)	50 (5.6 %)	18 (7.3 %)	0.61 (0.30–1.24)
Mean years	72.8	73.8	
Sex			
Female (0.13 %)	530 (59.6 %)	121(48.8 %)	1.57 (1.16–2.13)
Male (0.10 %)	359 (40.4 %)	127 (51.2 %)	1
Insured region			
Taipei city	137 (15.4 %)	61 (24.6 %)	1
Kaohsiung city	71 (8 %)	14 (5.6 %)	2.38 (1.24–4.57)
Northern Taiwan	200 (22.5 %)	58 (23.4 %)	1.65 (1.07–2.54)
Middle Taiwan	209 (23.5 %)	32 (12.9 %)	3.00 (1.83–4.90)
Southern Taiwan	240 (27 %)	78 (31.5 %)	1.39 (0.91–2.12)
Eastern Taiwan	21 (2.4 %)	4 (1.6 %)	2.48 (0.80–7.64)
Outlying island	11 (1.2 %)	1 (0.4 %)	4.98 (0.61–40.51)
Insured amount (NT$^d^)			
0	353 (39.7 %)	94 (37.9 %)	0.99 (0.68–1.45)
1–19,999	486 (54.7 %)	140 (56.5 %)	1
20,000–39,999	32 (3.6 %)	8 (3.2 %)	0.90 (0.38–2.15)
≥400,000	18 (2 %)	6 (2.4 %)	0.77 (0.28–2.13)
Numbers of anti-Alzheimer’s or BPSD drugs			
1	28 (3.1 %)	8 (3.2 %)	1
2	145 (16.3 %)	51 (20.6 %)	0.81 (0.35–1.90)
3	318 (35.8 %)	87 (35.1 %)	1.04 (0.46–2.37)
≥4	398 (44.8 %)	102 (41.1 %)	1.12 (0.49–2.52)
Numbers of dementia BPSD^e^			
0	102 (11.5 %)	46 (18.5 %)	1
1	122 (13.7 %)	61 (24.6 %)	0.90 (0.57–1.44)
Delirium	4	3	
Delusions	13	7	
Depression	18	12	
Behavioral disturbance	4	2	
Sleep disturbances	83	37	
Hallucination	0	0	
2	250 (28.1 %)	58 (23.4 %)	1.94 (1.24–3.05)
Delirium + delusions	8	7	
Delirium + depression	2	1	
Delirium + behavioral	0	0	
Delirium + sleep	12	6	
Delirium + hallucination	0	0	
Delusions + depression	10	6	
Delusions + behavioral	0	0	
Delusions + sleep	21	11	
Delusions + hallucination	0	0	
Depression + behavioral	1	0	
Depression + sleep	21	8	
Depression + hallucination	0	1	
Behavioral + sleep	6	0	
Behavioral + hallucination	0	0	
Sleep + hallucination	169	22	
≥3	415 (46.7 %)	83 (33.5 %)	2.26 (1.48–3.43)
Numbers of comorbidity			
0	25 (2.81 %)	10 (4.03 %)	1
1	74 (8.32 %)	27 (10.89 %)	1.1 (0.47–2.58)
2	148 (16.7 %)	65 (26.21 %)	0.91 (0.41–2.01)
≥3	642 (72.2 %)	146 (58.87 %)	1.76 (0.83–3.74)

^a^ CM refers to Chinese medicine

^b^ aOR refers to adjust odds ratio

^c^ CI refers to confidence interval

^d^ NT$ refers to new Taiwan dollars, of which 1 US $ = 30 NT$

^e^ BPSD refers to behavioral and psychological symptoms of dementia

Analyses of the major disease categories for all CM visits are summarized in Table 2. These results show that 18,138 (73.3 %) visits were treated with prescribed CHPs, whereas the remaining visits were treated with acupuncture and traumatology manipulative therapies. AD patients tended to use CHPs rather than acupuncture. “Symptoms, signs and ill-defined conditions” was cited as the most common reason for using CM (20.2 %, 5011 visits), followed sequentially by “diseases of the musculoskeletal system and connective tissue” (20.0 %, 4962 visits), “diseases of the respiratory system” (14.3 %, 3542 visits) and “diseases of the digestive system” (12.2 %, 3024 visits). Of the AD patients evaluated in the current study, 9.3 % sought CMs with the aim of treating their “mental disorders, diseases of the nervous system and sense organs”.

**Table 2:** Frequency distribution of Chinese medicine visits by major disease categories (according to 9th ICD codes) in Alzheimer’s patients from 1997 to 2008 in Taiwan

Major disease category	ICD-9-CM code range	Number of visits (%)		
		Chinese herbal remedies	Acupuncture, or manipulative therapies	Total of CM
Infectious and parasitic diseases	001–139	42 (0.2)	5 (0.02)	47 (0.2)
Neoplasms	140–239	119 (0.5)	0 (0)	119 (0.5)
Endocrine, nutritional, blood and metabolic diseases, and immunity disorders	240–289	799 (3.2)	25 (0.1)	824 (3.3)
Mental disorders, diseases of the nervous system and sense organs	290–389	1901 (7.7)	389 (1.6)	2290 (9.3)
Diseases of the circulatory system	390–459	1006 (4.1)	162 (0.7)	1168 (4.7)
Diseases of the respiratory system	460–519	3449 (13.9)	93 (0.4)	3542 (14.3)
Diseases of the digestive system	520–579	2954 (11.9)	70 (0.3)	3024 (12.2)
Diseases of the genitourinary system	580–677	789 (3.2)	40 (0.2)	829 (3.3)
Diseases of the skin and subcutaneous tissue	680–709	327 (1.3)	9 (0.04)	336 (1.4)
Diseases of the musculoskeletal system and connective tissue	710–739	2016 (8.1)	2946 (11.9)	4962 (20.0)
Symptoms, signs, and ill-defined conditions	780–799	3877 (15.7)	1134 (4.6)	5011 (20.2)
Injury and poisoning	800–999	77 (0.3)	852 (3.4)	929 (3.8)
Supplementary classification	V01–V82, E800–E999	0 (0)	4 (0.02)	4 (0.02)
Others^a^		322 (1.3)	848 (3.4)	1170 (4.7)
Total		18,138 (73.3)	6613 (26.7)	24,751 (100.0)

^a^ Other includes ICD-9-CM code range 740–779 and missing/error data

Table 3 shows the details of the CHPs most frequently prescribed by CM practitioners for treating mental disorders and diseases of the nervous system. The results show that *Bu*-*Zhong*-*Yi*-*Qi*-*Tang* was the most frequently prescribed CHP, followed by *Ji*-*Sheng*-*Shen*-*Qi*-*Wan*, *Ma*-*Zi*-*Ren*-*Wan* and *Tian*-*Wang*-*Bu*-*Xin*-*Dan*. The potential effects of the herbs contained in the 10 most common formulae are shown in Table 4. It is noteworthy that several herbs, including *Ginseng Radix(Ren*-*Shen)* and *Angelicae Sinensis Radix Integra(Dang*-*Gui)*, appeared repeatedly in formulae reported to be effective against AD. Table 5 shows that 77.1 % of the AD patients consumed *Bu*-*Zhong*-*Yi*-*Qi*-*Tang* during the period covered by the current study. After adjusting for demographic factors, the results revealed that female AD patients (OR = 1.23, 95 % CI 1.04–1.57) were more likely to consume *Bu*-*Zhong*-*Yi*-*Qi*-*Tang* than male patients.

**Table 3:** Ten most commonly used herbal formulae prescribed by CM practitioners for treating mental disorders, diseases of the nervous system and sense organs (total prescriptions: 1901)

Herbal formulae	English name	Average formulae dose (g/day)	*Ren*-*Shen* CHF dose (g/day)	*Ren*-*Shen* herbs (g/day)	*Dang*-*Qui* CHF dose (g/day)	*Dang*-*Qui* herbs (g/day)	Frequency of prescriptions (%)
*Bu*-*Zhong*-*Yi*-*Qi*-*Tang*	Center-supplementing Qi-boosting decoction	5.3	1.4	8.0	0.9	5.6	629 (33.1)
*Ji*-*Sheng*-*Shen*-*Qi*-*Wan*	Life saver kidney Qi Pill	5.1	–	–	–	–	538 (28.3)
*Ma*-*Zi*-*Ren*-*Wan*	Cannabis fruit pill	4.8	–	–	–	–	511 (26.9)
*Tian*-*Wang*-*Bu*-*Xin*-*Dan*	Celestial emperor heart-supplementing elixir	4.2	0.7	4.0	1.3	8.1	476 (25)
*Gan*-*Mai*-*Da*-*Zao*-*Tang*	Licorice, wheat, and jujube decoction	3.9	–	–	–	–	421 (22.1)
*Sheng*-*Mai*-*San*	Pulse-engendering powder	2.6	1.5	8.6	–	–	396 (20.8)
*Tian*-*Ma*-*Gou*-*Teng*-*Yin*	Gastrodia and uncaria beverage	5.5	–	–	–	–	381 (20)
*Liu*-*Wei*-*Di*-*Huang*-*Wan*	Six-ingredient rehmannia pill	2.1	–	–	–	–	336 (17.7)
*Qi*-*Bao*-*Mei*-*Ran*-*Dan*	Seven-jewel beard-blackening elixir	3.5	–	–	0.9	5.6	284 (14.9)
*Tao*-*Hong*-*Si*-*Wu*-*Tang*	Peach Kernel and Carthamus four agents decoction	2.2	–	–	0.4	2.5	258 (13.6)

**Table 4:** Potential effects of the herbs contained in the ten most common formulae prescribed by CM practitioners for treating Alzheimer’s disease

Herbal formulae	Constituents
*Bu*-*Zhong*-*Yi*-*Qi*-*Tang* ^a, b, c, d^	*Astragali Radix Cruda (Huang*-*qi),Ginseng Radix (Ren*-*shen)* ^a, b, c, d^ *, Glycyrrhizae Radix cum Liquido Fricta (Zhi*-*gan*-*cao), Atractylodis MacrocephalaeRhizoma (Bai*-*zhu), Angelicae Sinensis Radix Integra (Dang*-*gui)* ^a, c, d^ *, CitriReticulatae Pericarpium (Chen*-*pi), Cimicifugae Rhizoma (Sheng*-*ma), Bupleuri Radix (Chai*-*hu), Zingiberis Rhizoma Recens (Sheng*-*jiang), Jujubae Fructus (Da*-*zao)*
*Ji*-*Sheng*-*Shen*-*Qi*-*Wan* ^a, c^	*Rehmanniae Radix (Di*-*huang), Poria cum (Fu*-*ling)* ^a, c^ *, Corni Fructus (Shan*-*zhu*-*yu), Dioscoreae Rhizoma (Shan*-*yao), Moutan Cortex (Mu*-*dan*-*pi), Alismatis Rhizoma (Ze*-*xie), Aconiti Radix Lateralis (Fu*-*zi), Cinnamomi Cortex (Rrou*-*gui), Achyranthis Bidentatae Radix (Huai*-*niu*-*xi), Plantaginis Semen (Che*-*qian*-*zi)*
*Ma*-*Zi*-*Ren*-*Wan* ^a, c^	*Cannabis Fructus (Ma*-*zi*-*ren), Rhei Radix et Rhizoma (Da*-*huang)* ^c^ *, Paeoniae Radix Alba (Bai*-*shao)* ^a^ *, Aurantii Fructus Immaturus (Zhi*-*shi), Magnoliae Officinalis Cortex (Hou*-*po), Armeniacae Semen (Xing*-*ren)*
*Tian*-*Wang*-*Bu*-*Xin*-*Dan* ^a, b, c, d^	*Rehmanniae Radix (Di*-*huang), Ginseng Radix (Ren*-*shen)* ^a, b, c, d^ *, Polygalae Radix (Yuan*-*zhi)* ^c^ *, Scrophulariae Radix(Xuan*-*shen), Platycladi Semen (Bai*-*zi*-*ren), Platycodonis Radix (Jie*-*geng), Asparagi Radix (Tian*-*men*-*dong), Salviae Miltiorrhizae Radix (Dan*-*shen)* ^c^ *, Ziziphi Spinosi Semen (Suan*-*zao*-*ren), Ophiopogonis Radix (Mai*-*men*-*dong), Poria cum PiniRadice (Fu*-*shen)* ^a, c^ *, Angelicae Sinensis Radix Integra (Dang*-*gui)* ^a, c, d^ *, Schisandrae Fructus (Wu*-*wei*-*zi)*
*Gan*-*Mai*-*Da*-*Zao*-*Tang*	*Glycyrrhizae Radix Cruda (Zhi*-*gan*-*cao),Jujubae Fructus (Da*-*zao), Tritici Semen (Xiao*-*mai)*
*Sheng*-*Mai*-*San* ^a, b, c, d^	*Ginseng Radix (Ren*-*shen)* ^a, b, c, d^ *,Ophiopogonis Radix (Mai*-*men*-*dong), Schisandrae Fructus (Wu*-*wei*-*zi)*
*Tian*-*Ma*-*Gou*-*Teng*-*Yin* ^a, c^	*Gastrodiae Rhizoma (Tian*-*ma)* ^c^ *, Uncariae Ramulus cum Uncis (Gou*-*teng)* ^a^ *, Haliotidis Concha (Shi*-*jue*-*ming), Gardeniae Fructus(Shan*-*zhi*-*zi), Scutellariae Radix (Huang*-*qin), Eucommiae Cortex (Du*-*zhong), Loranthiseu Visci Ramus (Sang*-*ji*-*sheng), LeonuriHerba (Yi*-*mu*-*cao), PolygoniMultiflori Caulis (Ye*-*jiao*-*teng), Achyranthis Bidentatae Radix (Huai*-*niu*-*xi), Poria cum Pini Radice (Fu*-*shen)* ^a, c^
*Liu*-*Wei*-*Di*-*Huang*-*Wan* ^c^	*Rehmanniae Radix (Di*-*huang), Poria cum (Fu*-*ling)* ^c^ *, CorniFructus (Shan*-*zhu*-*yu), DioscoreaeRhizoma (Shan*-*yao), Moutan Cortex (Mu*-*dan*-*pi), AlismatisRhizoma (Ze*-*xie)*
*Qi*-*Bao*-*Mei*-*Ran*-*Dan* ^a, c, d^	*PolygoniMultiflori Radix (He*-*shou*-*wu), AchyranthisBidentatae Radix (Huai*-*niu*-*xi), AngelicaeSinensis Radix Integra (Dang*-*gui)* ^a, c, d^ *, Poria cum (Fu*-*ling), Cuscutae Semen (Tu*-*si*-*zi), LyciiFructus (Gou*-*qi*-*zi), PsoraleaeFructus (Po*-*gu*-*zhi)*
*Tao*-*Hong*-*Si*-*Wu*-*Tang* ^a, c, d^	*Persicae Semen (Yao*-*ren), Carthamustinctorius Flos (Hong*-*hua), Angelicae Sinensis Radix Integra (Dang*-*gui)* ^a, c, d^ *, Chuanxiong Rhizoma (Chuan*-*xiong), Paeoniae Radix Alba (Bai*-*shao)* ^a^ *, Rehmanniae Radix (Di*-*huang)*

^a^ Formula containing a single herb that might promote the neurotransmitter acetylcholine

^b^ Formula containing a single herb that might inhibit the *N*-methyl-D-aspartate (NMDA) receptor

^c^ Formula containing a single herb that might reduce the *β*-amyloid

^d^ Formulae containing a single herb that might decrease the Tau protein

**Table 5:** Demographic characteristics of *Bu*–*Zhong*–*Yi*–*Qi*–*Tang* use among patients with Alzheimer’s disease

Characteristics	*Bu*–*Zhong*–*Yi*–*Qi*–*Tang* users (%)	CM^a^ users not using *Bu*–*Zhong*–*Yi*–*Qi*–*Tang* (%)	aOR^b^ (95 % CI^c^)
No. of cases	685 (77.1)	204 (22.9)	
Age at diagnosis			
<45	5 (0.7)	2 (1)	0.69 (0.12–6.03)
45–54	21 (3.1)	2 (1)	3.17 (0.70–17.44)
55–64	88 (12.8)	22 (10.8)	1
65–74	221 (32.1)	70 (34.3)	0.72 (0.23–1.34)
75–84	304 (44.1)	92 (45.1)	0.92 (0.41–1.52)
≥85	45 (6.5)	16 (7.8)	0.64 (0.22–1.59)
Sex			
Female	377 (55.0)	99 (48.5)	1.23 (1.04–1.57)
Male	308 (45.0)	104 (51.0)	1
Numbers of anti–Alzheimer’s or BPSD drugs			
1	26 (3.8)	11 (5.4)	1
2	133 (19.4)	55 (27.0)	0.82 (0.21–2.01)
3	259 (37.8)	75 (36.8)	1.32 (0.52–2.81)
≥4	367 (39.0)	63 (40.9)	1.04 (0.57–2.11)
Numbers of dementia BPSD^d^			
0	84 (12.3)	42 (20.6)	1
1	115 (16.8)	63 (30.9)	0.72 (0.31–1.68)
2	206 (30.1)	52 (25.5)	2.11 (0.93–3.38)
≥3	280 (40.9)	63 (30.9)	2.75 (0.82–4.31)
Numbers of comorbidity			
0	22 (3.2)	12 (5.9)	1
1	90 (13.1)	35 (17.2)	1.21 (0.38–3.71)
2	119 (17.4)	57 (27.9)	1.02 (0.31–4.23)
≥3	454 (66.3)	99 (48.5)	2.24 (0.71–4.55)

^a^ CM refers to traditional Chinese medicine

^b^ aOR refers to adjust odds ratio

^c^ CI refers to confidence interval

^d^ BPSD refers to behavioral and psychological symptoms of dementia

## Discussion

To the best of our knowledge, this study was the first reported one using a random national-level sample to document the usage characteristics of traditional Chinese medicines in AD patients. The incidence of AD was less than 0.3 % in people aged 55–64 years, with the incidence increasing with increasing age. In people aged 85 years or older, the incidence was found to be 17.9 %, which is low compared with results of previous surveys [10, 11]. Notably, all of the patients described in the current study were newly diagnosed with AD by board-certified neurologists or psychiatrists and selected from a random sample of one million patients. Furthermore, these patients were selected from the insured general population of Taiwan and the rate of insured people was consistently above 97 % since 1997. The possibility of selection or recall bias could therefore be excluded.

More than 78 % of the AD patients in the cohort were used some form of CM during the 12 years covered by the current study. High accessibility and full coverage for CM through the NHI scheme in Taiwan could lead to an increase in CM use. Furthermore, several Chinese herbs may be effective as alternative therapies for treating AD and related disorders [12]. The current findings also show that women and those living in urban areas are more likely to use CM than men and patients living rural areas. Similar trends were reported previously [13].

BPSD relating to AD such as delusions, paranoia, hallucinations and anxiety could lead to an increase in caregiver burden and accelerate the progression of AD [14]. AD patients suffering from one or more BPSD were more likely to use traditional CM than those with no BPSD. In addition, using anti-AD or BPSD drugs did not lead to a reduction in CM use. AD patients consumed herbal therapies with the aim of relieving their AD-related symptoms, rather than using these treatments as alternatives to standard anti-AD treatments (i.e., herbal therapies were consumed as complementary medicines).

The category “symptoms, signs and ill-defined conditions” was the most frequently cited diagnosis in the disease category for CM visits to classify the diagnosis pattern of CM. This phenomenon has also been observed in several other patient populations [15]. In addition, 9.3 % of patients with AD in the “mental disorders, diseases of the nervous system and sense organs” disease category had specifically sought out CM to treat their condition. CM treatments were generally used as adjuvant therapies to relieve AD-related symptoms rather than being used as replacements for conventional anti-AD treatment.

The category “diseases of the digestive system” was cited as one of the most common reasons for using CHPs (11.9 %, 2954 visits), with *Ma*-*Zi*-*Ren*-*Wan* being used to treat constipation [16]. Constipation is a common problem among geriatric patients, especially those with AD, in whom it is generally exacerbated by a lack of exercise or the side effects of anti-AD drugs [17]. Severe constipation in AD patients can lead to confusion and symptoms of irritability or aggression because of the pain and discomfort associated with this condition [18]. Moreover, emodin, which is the major chemical constituent of *Ma*-*Zi*-*Ren*-*Wan*, protects the cortical neurons from *β*-amyloid-induced toxicity, representing a possible mechanism for treating AD [19]. In this study, *Bu*-*Zhong*-*Yi*-*Qi*-*Tang* was determined to be the most commonly prescribed formula, especially for female AD patients. *Bu*-*Zhong*-*Yi*-*Qi*-*Tang* led to elevated levels of dopamine and noradrenaline in the cortical tissues of mice, as well as improved attention, learning function and memory [20, 21]. *Bu*-*Zhong*-*Yi*-*Qi*-*Tang* was also used to treat elderly patients with weakness and fatigue [22].

Some frequently prescribed formulae have been reported to exhibit potentially positive effects on AD. For example, *Sheng*-*Mai*-*San* was reported to improve memory and decrease neuronal apoptosis in the hippocampus [23]. Furthermore, *Ji*-*Sheng*-*Shen*-*Qi*-*Wan* and *Liu*-*Wei*-*Di*-*Huang*-*Wan*, which both belong to the *Di*-*Huang*-*Wan* group of formulae and share similar herbal components, were reported to increase cognitive function after 2 months of treatment [24]. Some formulae were reported to show neuroprotective activity. For example, *Tian*-*Ma*-*Gou*-*Teng*-*Yin* led to a reduction in necrosis and apoptosis in neuronal cells through a variety of pharmacological effects, including anti-inflammatory, antioxidative and antiapoptotic activities [25]. *Tao*-*Hong*-*Si*-*Wu*-*Tang* exhibited neuroprotective effects against focal cerebral ischemia. Moreover, *Gan*-*Mai*-*Da*-*Zao*-*Tang* exhibited sedative and anti-anxiety effects in an animal model [26]. All of the aforementioned formulae, which are standard formulae recommended by the DCMP, Ministry of Health and Welfare, Taiwan, must be modified by qualified CM practitioners in accordance with the principle of syndrome pattern identification as the basis for determining treatment.

Table 4 lists all of the herbs contained in the top ten most frequently prescribed formulae. *Ginseng Radix* (*Ren*-*Shen*), which is the major constituent herb in three of the top ten formulae, has been reported to show multiple effects against AD-related factors such as decreasing *β*-amyloid formation [27], increasing choline acetyltransferase [28] and forming a competitive interaction with the *N*-methyl-*D*-aspartate receptor [29]. *Ginseng Radix* has also been reported to increase cognitive function [30]. *Angelicae Sinensis Radix Integra* (*Dang*-*Gui*) is the key component of *Bu*-*Zhong*-*Yi*-*Qi*-*Tang*, *Tian*-*Wang*-*Bu*-*Xin*-*Dan* and *Tao*-*Hong*-*Si*-*Wu*-*Tang*. An ethanolic extract of *Dang*-*Gui* enhanced cholinergic function [31], inhibited acetylcholinesterase [32], reduced *β*-amyloid associated neurotoxicity and decreased Tau protein hyperphosphorylation in a dose-dependent manner [33].

There were several limitations associated with the current study. First, health foods containing herbs, folk medicines and prescriptions from CM practitioners without a license from the Taiwanese authorities were not included in this study. Therefore CHP use may have therefore been underestimated. However, high healthcare insurance coverage and low prices of government-approved CM led to a reduction in herbal folk medicine use. Second, it is not possible to draw any conclusions regarding the relationship between cognitive function and CM use because of a general lack of detailed clinical data. This study focused primarily on a patient population with AD, the AD diagnosis was rigorously censored by the Bureau of National Health Insurance. Third, this study was conducted retrospectively and did not include a randomized placebo group. Lastly, the CM formulae were modified according to the principle of syndrome differentiation.

## Conclusions

Most people with AD who consumed herbal products used supplement *qi*, nourish the *blood*, and quiet the heart spirit therapy as complementary medicines to relieve AD-related symptoms, in addition to using standard anti-AD treatments.

## Additional files

10.1186/s13020-016-0086-9 The approval Letter from Taipei City Hospital Institutional Review Board.
10.1186/s13020-016-0086-9 The approval Letter from National Institutes of Health.

## Abbreviations

AD: Alzheimer’s disease
CM: Chinese medicine
CHPs: Chinese herbal products
BPSD: behavioral and psychological symptoms of dementia
NHIRD: National Health Insurance Research Database
NHI: National Health Insurance
ICD-9-CM: international classification of diseases, ninth revision, clinical modification
NINCDS-ADRDA: National Institute of Neurological and Communicative Disorders and Stroke-Alzheimer’s Disease and Related Disorders Association
DSM IV: diagnostic and statistical manual of mental disorders
DCMP: Department of Chinese medicine and pharmacy
GMP: good manufacturing practice
MMSE: mini-mental state examination
