# Supplementary material for: Patterns of Chinese medicine use in prescriptions for treating Alzheimer’s disease in Taiwan
Source: Chin Med. 2016 Mar 28;11:12. doi: 10.1186/s13020-016-0086-9 (PMC4810525; doi:10.1186/s13020-016-0086-9)
Supplement: Supplementary file 1 — 10.1186/s13020-016-0086-9 The approval Letter from Taipei City Hospital Institutional Review Board. [file 13020_2016_86_MOESM1_ESM.pdf]

臺北市立聯合醫院人體試驗委員會
Taipei City Hospital Institutional Review Board

計畫執行許可書

中華民國 102 年 9 月 13 日
聯絡人：高凡
電話:(02)27093600 轉 3828
傳真:(02)27079021

案件編號：TCHIRB-1020816-E
計畫名稱：以健保資料庫分析臺灣失智患者中西藥使用情形
計畫主持人：林舜穀(仁愛院區)
計畫書版本/日期:20130911Ver2
同意書版本/日期:免受試者同意書
計畫期間為 102 年 10 月 01 日至 103 年 12 月 31 日
上述計畫於 102 年 9 月 12 日審查通過，於 102 年 10 月 16 日本院一百零二年第十次人體試驗委員會會議追認核備，特此證明。

本許可書有效期限自民國 102 年 9 月 12 日起至民國 103 年 9 月 11 日止。

※追認核備之案件，若會議有疑義時，本會有權撤銷此計畫執行許可書。
※未完成期中報告或結案報告者不得申請新案。
※請於計畫執行許可書有效期限到期前一個月內繳交期中報告。
※請於計畫執行許可書到期前繳交結案報告。
※計畫內容若有任何修改或增減，計畫主持人或計畫委託單位應檢送修正案至本會，經審查通過後方可實施。

田麗珠 田麗珠

臺北市立聯合醫院人體試驗委會

CERTIFICATE

Date：September 13, 2013

The project entitled "Prescription Pattern of Chinese and Western Medicine for Dementia Patients in Taiwan with Nation Health Insurance Research Database"submitted by investigator Shun-Ku,Lin has been approved by Institutional Review Board of the Taipei City Hospital.
Protocol Version Date:20130911Ver2
ICF Version Date:Waiver
Study period: since2013/10/01 to 2014/12/31
Above project is approved by the TCHIRB on2013/9/12 and valid till 2014/9/11.

※ All protocols should been subject to final endorsement by the board. TCHIRB has the right to revoke the approval.
※ No new protocol can be applied if the midterm report or the final report is not handed in.
※ The midterm report should be handed in one month before the expiry date of this certificate.
※ The final report should be handed in before the expiry date of this certificate or project end.
※ The PI should send the amendments to TCHIRB if there are changes or modification related the protocol. Any change should not be executed until being approved by TCHIRB.

Lih-Chu Tien
Lih-Chu Tien
Chairman
Taipei City Hospital Institutional Review Board

臺北市立聯合醫院人體試驗委員會

The Committee is Organized and operates in accordance with ICH-GCP regulations and guideline.
本委員會組織與運作皆遵守 ICH-GCP 規定

臺北市立聯合醫院人體試驗委員會
Taipei City Hospital Institutional Review Board

計畫主持人您好:

本會近來追蹤審查研究計畫案發現數起試驗偏離狀況，在此特別提醒您留意以下事項:

一、 同意書取得及簽署：

1. 請務必使用本會核准之同意書版本給予受試者簽署。
2. 受試者之招募必需於收到本會之計畫執行許可書後，始可為之。
3. 受試者同意書務必讓受試者簽署並填寫日期。
4. 同意書中計畫主持人需親自簽名並填寫日期。
5. 試驗相關人員不得為見證人。
6. 簡易案件若計畫期限為一年期或一年以內之案件，只需繳交結案報告。若為多年期計畫，原則每年繳交期中報告，但有疑義之案件，委員可提會討論決議繳交期中報告之頻次。
7. 一般案件依影響受試者權益、安全、福祉於會議上討論並決議繳交期中報告之頻次。
8. 請於計畫執行許可書有效期限到期前一個月內繳交期中報告。未依規定繳交期中報告者，許可書失效期間應暫停納入新受試者;未繳交期中報告者不得申請新案並暫停審理中之案件，直到該期中報告審查通過。
9. 後續追蹤審查發現任何影響受試者權益、安全、福祉之案件，會於會議上討論並決議是否更改繳交期中報告之頻次。
10. 請於計畫執行許可書到期前繳交結案報告。未繳交結案報告者不得申請新案並暫停審理中之案件，直到該結案報告審查通過。

二、 變更各項已通過之資料需檢送修正案至本會審查同意後執行。

以上務必請主持人遵守相關規定，以免增加不必要之困擾。

臺北市立聯合醫院 TPECH 人體試驗委員會

人體試驗委員會 敬上

臺北市立聯合醫院人體試驗委員會
Taipei City Hospital Institutional Review Board

97 年 8 月 27 日制定
98 年 12 月 16 日修訂
101 年 8 月 15 日修訂

主持人您好:

您申請本人體試驗委員會審查的案件已通過審查並發予計畫執行許可書。為符合『人體研究各項法規及本會 SOP 之規定』下列之事項請您特別注意並配合實施。

1. 請依本會核可之計畫書及受試者同意書版本確實執行，並確實做到知情同意及受試者隱私保護。
2. 招募受試者進行方式應適當，於計畫執行期間及完成後，受試者的隱私都應受到保護。
3. 當研究涉及易受傷害族群時，如小孩、孕婦、受刑人....等，應給予適當的安全保護，以確保他們的權利與福祉。
4. 於計畫執行期間，受試者應可獲得相關必要的資訊；並受到適當的醫療照護；當受試者自願退出時仍應受適當的待遇與治療。
5. 對受試者因參與計畫受到傷害時之補償應適當。
6. 當有任何新資訊可能不利受試者的安全時，研究人員需立即呈報本委員會。
7. 計畫主持人或計畫委託單位若知道有任何修改或增添計畫之內容，應申請修正並再次獲得本委員會之審查通過後方可實施。(修正案申請文件請至院外網站>>人體試驗委員會>修正案下載) 。
8. 當您計畫執行時發生死亡、危及生命、導致病人住院或延長病人住院等疑似未預期嚴重不良反應(Suspected Unexpected Serious Adverse Reaction, SUSAR) 應立即呈報本委員會 (SUSAR 通報請至院外網站>人體試驗委員會> SUSAR 下載所需文件)。
9. 期中及結案報告之繳交:

(1) 簡易案件若計畫期限為一年期或一年以內之案件，只需繳交結案報告。若為多年期計畫，原則每年繳交期中報告，但有疑議之案件，委員可提會討論決議繳交期中報告之頻次。

(2) 一般案件依影響受試者權益、安全、福祉於會議上討論並決議繳交期中報告之頻次。

(3) 請於計畫執行許可書有效期限到期前一個月內繳交期中報告。未依規定繳交期中報告者，許可書失效期間應暫停納入新受試者;未繳交期中報告者不得申請新案並暫停審理中之案件，直到該期中報告審查通過。

(4) 後續追蹤審查發現任何影響受試者權益、安全、福祉之案件，會於會議上討論並決議是否更改繳交期中報告之頻次。

(5) 請於計畫執行許可書到期前繳交結案報告。未繳交結案報告者不得申請新案並暫停審理中之案件，直到該結案報告審查通過。

相關文件請至院外網站>人體試驗委員會>期中報告；院外網站>人體試驗委員會>結案報告 下載。

計畫執行許可書接收回函

案件編號: TCHIRB-1020816-E

一、本人已收到臺北市立聯合醫院人體試驗委員會所核發之計畫執行許可書並確認無誤。

二、以上述注意事項資料本人已詳細閱畢，並願意遵守所有規定，若有違反願意接受貴會的所有罰則。

計畫主持人: [illegible]　　日期: 102 年 9 月 18 日

※此回函請主持人確實以傳真方式簽回，傳真: 02-27079021，若本會未收到本回函表示主持人未同意上述注意事項。

臺北市立聯合醫院 人體試驗委員會 TPECH
