# Supplementary material for: Patterns of Chinese medicine use in prescriptions for treating Alzheimer’s disease in Taiwan
Source: Chin Med. 2016 Mar 28;11:12. doi: 10.1186/s13020-016-0086-9 (PMC4810525; doi:10.1186/s13020-016-0086-9)
Supplement: Supplementary file 2 — 10.1186/s13020-016-0086-9 The approval Letter from National Institutes of Health. [file 13020_2016_86_MOESM2_ESM.pdf]

附件二

全民健康保險研究資料庫資料加值服務
繳 費 通 知 單

致 賴榮年 助理教授 (NHIRD-103-091)

一、台端申請「全民健康保險研究資料庫」相關資料，經諮詢確認本案提供下列光碟片編號：R201_1996~R225_1996、R201_1997~R225_1997、R201_1998~R225_1998、R201_1999~R225_1999、R201_2000~R225_2000、R201_2001~R225_2001、R201_2002~R225_2002、R201_2003~R225_2003、R201_2004~R225_2004、R201_2005~R225_2005、R201_2006~R225_2006、R201_2007~R225_2007、R201_2008~R225_2008、R201_2009~R225_2009、R201_2010~R225_2010、R201_2011~R225_2011、R201-2012~R225_2012 共計 425 片光碟片（每片光碟片 500 元），合計新台幣 212,500 元（每片光碟片 500 元）。

二、於收到本通知後，請儘速將所需費用利用各大行庫匯款或 ATM 轉帳至
台新銀行---新生分行（銀行代號：812）
帳號：００４－１０－０７０５６０－８－００
戶名：財團法人國家衛生研究院

三、於匯款後，擬請申請人務必盡速將請將匯款單影本及本通知單，傳真至 (037) 586-410，國衛院生醫資源中心收。本院在確認收到本項費用後 14 個工作日內，即以掛號寄交所核給之資料及繳款發票。

國家衛生研究院
生醫資源中心　　敬啟

財團法人國家衛生研究院 生醫資源中心

民國 103 年 4 月 22 日

回條：

1. 本人確認申請項次一所列資料無誤，並支付所列費用（匯款單如附）。

2. 請開立發票 ☑三聯式抬頭：＿＿＿＿＿＿＿＿統一編號：＿＿＿＿＿＿＿
　　　　　　 ☐二聯式抬頭：＿＿＿＿＿＿＿＿

3. 請勾選欲儲存資料之媒體 ☐CD 片 ☐DVD 片 ☑硬碟(由申請人提供予本院)

※ 請注意：請務必於付款前、或開立發票前，請確認 貴單位核銷之規定。

申請人簽章：[illegible]

填寫日期：103 年 4 月 24 日

承辦人：孫憲萍 ☎037-246166 分機 33601

全民健康保險研究資料庫資料加值服務
專家諮詢通知書

申請者姓名：賴榮年　　　　　　　　　編號：NHIRD-103-091

壹、建議事項

壹、　本案依專家建議提供資料。

貳、　資料內容之相關資訊，請參閱網站。網址：http://nhird.nhri.org.tw/

貳、建議申請 光碟如下

增加		減少	

建議申請光碟片總清單

R201_1996~R225_1996 、 R201_1997~R225_1997 、 R201_1998~R225_1998 、
R201_1999~R225_1999 、 R201_2000~R225_2000 、 R201_2001~R225_2001 、
R201_2002~R225_2002 、 R201_2003~R225_2003 、 R201_2004~R225_2004 、
R201_2005~R225_2005 、 R201_2006~R225_2006 、 R201_2007~R225_2007 、
R201_2008~R225_2008 、 R201_2009~R225_2009 、 R201_2010~R225_2010 、
R201_2011~R225_2011、R201-2012~R225_2012 共 425 片光碟片

承辦人：孫憲萍 (037)246-166 分機 33601

財團法人國家衛生研究院
生醫資源中心
苗栗縣竹南鎮350科研路[illegible]

附件三

適用政府採購法機關辦理採購之注意事項

1. 國家衛生研究院（以下簡稱本院）提供之「全民健康保險研究資料庫」（以下簡稱本資料庫）資料加值服務，係受中央健康保險署（以下簡稱健保署）委託執行「全民健康保險研究資料庫委託建置及管理」案；本案經健保署公開招標，由本院得標。因本案採非複數決標，故本院為唯一得標之機構。
2. 本資料庫係在保護個人隱私前提下，依照「電腦處理個人資料法」第八條及第十八條開放學術研究使用，非屬一般商品，申請人應遵守事項如下：

   (1) 申請人須依「國家衛生研究院全民健康保險研究資料庫資料加值服務申請原則」提出申請，申請案須經專家諮詢後，本院方得提供。

   (2) 申請人須簽署「使用同意書」，並確實遵守相關規定，所申請之加值資料限於所申請之研究主題使用，且不得因任何理由辨識或揭露個人或個別機構；如有違反，本院得依「國家衛生研究院全民健康保險研究資料庫資料加值服務申請原則」第十二條，要求立即繳回所申請加值資料，且不得保留任何備份或相關加值資料檔案，國衛院並得停止其個人或單位申請及使用本加值資料之權利，並保留法律責任之追訴權。
3. 本加值資料庫內容來自健保署，本院無權刪改，且與資料內容正確性無涉。申請人應詳細了解本資料庫之性質與內容，對其研究使用自負全責，且不得以本資料庫內容或所提供之資訊主張任何權利及作為任何法律上求償或訴訟之依據。
4. 本資料庫依使用者付費之原則，酌收加值服務資料處理工本費用，包括所需人力、機器操作時間等費用，收費標準係由健保署同意後實施，並公告於網頁，無法減價。
5. 申請案均先繳款後開發票，繳款後方進行加值服務資料處理，於雙方協議時間內取件。凡需驗收後付款者，驗收標的得為所申請加值資料清單或擷取條件確認等相關文件。
6. 特殊需求申請案加值資料儲存媒體須由申請人提供媒體存放，儲存媒體費用由買方負擔。
7. 申請案適用採購法第三十條各款，本院免付押標金、履約保證金。
